# Mendelian randomization explores the causal relationships between obesity, diabetes, inflammation and nonalcoholic fatty liver disease

**DOI:** 10.1097/MD.0000000000034638

**Published:** 2023-09-22

**Authors:** Xing Wang, Dianpeng Zhao, Lichao Cheng, Jing Gao, Jian Li, Chao Geng

**Affiliations:** a Department of Hepatobiliary and Pancreatic Surgery, Affiliated Hospital of Weifang Medical University, Weifang, P.R. China.

**Keywords:** mendelian randomization, obesity, diabetes, NAFLD

## Abstract

Nonalcoholic fatty liver disease (NAFLD) is one of the most common chronic liver diseases worldwide. Observational studies have revealed various risk factors associated with NAFLD, while the causal relationships between NAFLD and clinical diseases (including obesity, diabetes and inflammation) remain unclear. In this study, based on the genome-wide association study (GWAS) data, a two-sample Mendelian randomization (MR) analysis was conducted to evaluate the causality between NAFLD and 6 clinical indicators, including body mass index (BMI), waist-to-hip ratio (WHR), C-reactive protein (CRP), fasting blood glucose (FG), fasting insulin (FI), and glycosylated hemoglobin (HbA1c). MR is based on Mendel’s law of inheritance, which uses genetic variation as a toll variable to affect the health of a population to infer causal effects in the presence of unobserved confounding. Inverse variance weighted method was the main MR method. In addition, we performed multiple steps of variable screening in the method to ensure that we were conducting the study under the MR assumption. In the MR analysis, a higher WHR (*P* = .0078; OR = 1.008; 95% CI, 1.002–1.013) was genetically predicted to be causally associated with an increased risk of NAFLD, while patients with higher HbA1c had a lower risk of NAFLD (*P* = .0437; OR = 0.44; 95% CI, 0.20–0.97). Our results showed that the genetically driven WHR and HbA1c might be potential causal factors for NAFLD, while BMI, FG, FI, and CRP were not causal factors for NAFLD, which explained the promoting role of WHR and HbA1c in the occurrence and development of NAFLD. Our finding hence revealed new insights into how nature and nurture factors underpin NAFLD, providing positive effect on the causes and prevention of this disease.

## 1. Introduction

Nonalcoholic fatty liver disease (NAFLD) is one of the most common chronic liver diseases worldwide, which represents a spectrum of the liver disease characterized by ectopic fat accumulation without other causes for secondary hepatic fat accumulation (e.g., excessive alcohol consumption).^[1–3]^ A systematic review reported that the prevalence of NAFLD has risen globally from 25.3% between 1990 to 2006 to 38.0% between 2016 to 2019.^[4]^ Furthermore, NAFLD can transform to more severe forms (nonalcoholic steatohepatitis, fibrosis and cirrhosis), leading to the occurrence of hepatocellular carcinoma (HCC).^[5]^ The incidence of HCC among non-cirrhotic NAFLD patients is approximately 0.1 to 1.3 per 1000 patient-years.^[6]^ According to the “multiple hit” hypothesis, multiple insults, such as insulin resistance, hormones secreted from the adipose tissue, and nutritional factors, might acting together on genetically predisposed subjects for the induction of NAFLD, providing a more precise elucidation of the pathogenesis of NAFLD.^[7]^ Therefore, it is urgent to uncover the pathogenic components of NAFLD.

Obesity is defined as an excess of body weight, and the prevalence of obesity has increased over the last several decades.^[8]^ Jarvis’s study suggested that obesity was independently associated with increase in risk of incident severe liver disease.^[9]^ In addition, It has been observed that obesity is the most effective predictors for NAFLD.^[10–13]^ However, reports on the impact of obesity on NAFLD are controversial.^[9,14,15]^ The relational between NAFLD and diabetes is bidirectional.^[16]^ The occurrence rate of NAFLD in individuals with type 2 diabetes, based on ultrasound or proton magnetic resonance spectroscopy, is about 55.48% worldwide (95% CI: 47.26–63.67), with the Europe harboring the highest prevalence (68.0% [62.1–73.0%]).^[17]^ In turn, the incidence of diabetes could be increased by more than 2 times owing to the NAFLD, with risk paralleling the underlying NAFLD severity.^[18]^ Paradoxically, Anita et al demonstrated that genetically-influenced insulin resistance by homeostasis model assessment does not increase the risk of NAFLD.^[19]^ Therefore, the specific indicator of diabetes which induces NAFLD is still unclear. The intimate association between diabetes and NAFLD has raised the hypothesis that NAFLD could not only be induced by diabetes but that it might also directly aggravate diabetes-related indicators and, subsequently, increase the risk of developing diabetes.^[20]^ Hyperglycemia in type 2 diabetes directly causes high hepatic uptake of glucose and subsequently contribute to hepatotoxicity induced by increased glucose metabolism and increased compensatory de novo lipid synthesis, which might induce the NAFLD.^[21]^ However, this master hypothesis has not yet been definitely proved and the specific indicator of diabetes which induces NAFLD remains uncertain. Fasting blood glucose (FG), Fasting insulin (FI), and glycosylated hemoglobin (HbA1c) are significant indicators in the clinical treatment of diabetes.^[22]^ In addition, C-reactive protein (CRP) is proved to be closely associated with the high risk of NAFLD.^[23]^ In this study, 2 obesity-related indicators, 3 diabetes-related indicators and one inflammation-related indicator were included to investigate the association between them and NAFLD.

Exploring the causal associations between clinical indicators and NAFLD is significant for investigating diseases predisposing factors and choosing clinical treatments. Observational study has certain limitations in identifying the causal associations, which can not eliminate the inference of confounding factors (e.g. socioeconomic status, lifestyle) on the experimental results or identify the direction of causation. To solve these problems, a novel approach called Mendelian randomization (MR) is proposed. The MR analysis, which uses one or more genetic variants as instrumental variables (IVs) for the risk factors of interest, has been widely used in exploring the causal associations between exposures (risk factors) and outcomes (human diseases or human disease phenotypes). For instance, Younossi et al^[24]^ ruled out the possibility that blood tyrosine levels could be a novel biomarker for NAFLD using MR design, challenging the traditional concept. Besides, the MR analysis conducted by Sehoon et al^[25]^ supports the causal reduction in kidney function by NAFLD, suggesting the necessity of kidney function protection of NAFLD patients. With the rapid development of genome-wide association study (GWAS) and more publicly GWAS datasets becoming available, MR design based on two-sample setting becomes a reliable and accessible method to conduct causal inference.

There is no researcher that examines the causal relationship between the 6 clinical indicators (including body mass index (BMI), waist-to-hip ratio (WHR), FG, FI, HbA1c, and CRP) and NAFLD. In this study, we used Mendelian randomization to systematically explore whether these 4 indicators causally increase the risk of incident NAFLD, aiming to provide new insight to the clinical treatment of NAFLD.

## 2. Materials and Methods

### 2.1. Data sources of exposures

The genetic variants with BMI were obtained from a GWAS meta-analysis which included about 700,000 participants of European ancestry with 2.3 million single nucleotide polymorphisms (SNPs) from GIANT, which was the largest and latest GWAS summary data for BMI. They performed a GWAS of BMI in UKB participants of European ancestry.^[26]^ The genetic variants with WHR were obtained from a GWAS meta-analysis in 694,649 individuals of European ancestry with 2.7 million SNPs combining UK Biobank and GIANT, which was the largest and latest GWAS summary data for WHR.^[27]^ The genetic variants with FG, FI were obtained from GWAS summary statistics for up to 151,188 individuals (FG meta-analysis) and 105,056 individuals (FI met-analysis), and these summary data in FG/FI GWAS were evaluated from individuals of European descent without diabetes within the Meta-Analysis of Glucose an Insulin-related traits Consortium.^[28]^ The genetic variants with HbA1c were from an ancestry-specific GWAS meta-analysis in up to 123,665 individuals of European ancestry.^[29]^ The genetic variants with CRP were obtained from a transethnic GWAS meta-analysis including 49,839 individuals.^[30]^

### 2.2. Data sources of outcome

The genetic associations with NAFLD were extracted from 2 independent GWAS summary statistics where 1 consists of 1106 European cases and 8571 European controls,^[31]^ and the other is composed of 1483 European cases and 17,781 European controls.^[32]^ The former NAFLD was determined by electronic medical records and genomics (eMERGE) network while the latter NAFLD was diagnosed by histopathology.

### 2.3. Selection of genetic variants mimicking exposures

Based on the GWAS results on obesity and lipid-related indices, we firstly selected SNPs that were strongly associated with interested exposures respectively at a genome-wide significant level of *P* < 5 × 10^−8^. In order to eliminate the effect of linkage disequilibrium (LD) on the study results, we identified the independent SNPs by clumping the top GWAS loci through PLINK 1.9 (https://www.cog-genomics.org/plink2) with the threshold of LD R2 < 0.1 in a 500kb window. The LD was based on the European samples of the 1000 Genomes Project.^[33]^ Weak IVs may induce estimator bias, so the F statistics of all genetic variables in the present study were larger than 10.

### 2.4. Mendelian randomization estimation

**Figure 1.**
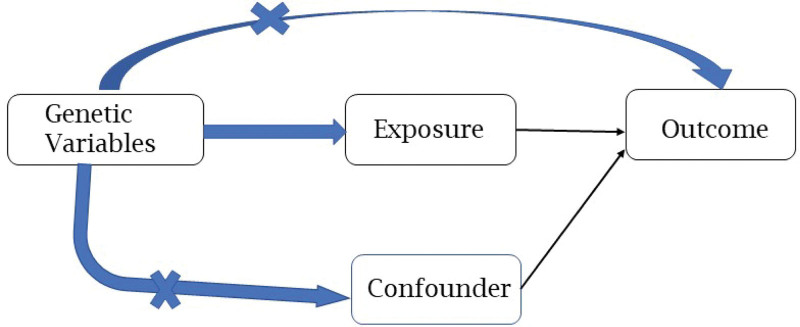
The diagram in Mendelian randomization. There were 3 assumptions when using Mendelian randomization: (A) Correlation hypothesis: genetic variants used as instrumental variables are associated with exposure; (B) Independence/exchange ability assumption: no common cause for genetic variation and target outcome; (C) Exclusively/no horizontal pleiotropy hypothesis: no independent causal pathway between genetic variation and outcome except through exposure.

For MR estimation, the basic diagram structure was showed in Figure 1. In general, 3 assumptions must be satisfied when using MR analysis. The first assumption is that the instrumental variables are associated with the exposure. The second one is that instrumental variants are not associated with observed or unobserved confounder factors. The third one is that instrumental variants affect outcome only through exposure.^[34]^ The MR analyses were performed by using publicly available, large-scale GWAS summary data.

There will be potential violations of assumptions 2 or 3 when conducting MR analysis. Specifically, when the instrumental variable has an effect on the outcome through other biology pathways not only through exposure. So, it is essential to assess the pleiotropy in an MR analysis. We used MR-PRESSO global test^[35]^ and Q’ statistics^[36]^ to identify horizontal pleiotropic outliers and quantify the heterogeneity. MR-PRESSO was used to identify pleiotropic outliers with a significance level of *P* < .05.

We used the inverse variance weighted (IVW) method as the main method to evaluate the total causal effect of the exposure on the outcome by assuming a fixed-effect model. IVW method minimized the weighted average variance by aggregating 2 or more IVs, where the IV’s weight was the inverse of the variance of the effect estimate.^[37]^ Besides the IVW method, we estimated the causal estimates using weighted median regression (WMR)^[38,39]^ and Egger regression method (MR Egger)^[40]^ as additional analyses. WMR can give a consistent estimator even when up to 50% invalid instrumental variables. MR-Egger can explore the average horizontal pleiotropic effect across IVs.

For the causal estimation, *P* value less than .05 was considered as statistically significant.MR analysis was performed with R package “MendelianRandomization” in R version 4.1.0 (http://www.r-project.org/).

## 3. Results

### 3.1. Mendelian randomization analyses between obesity traits and nonalcoholic fatty liver disease

We used 690 genetic variants as the instrumental variables with which to perform the MR study between BMI and NAFLD from eMERGE network. The IVW method results (*P* = .7356; OR = 0.94; 95% CI, 0.68–1.31) indicated there was no causal relationship between BMI and NAFLD (Table 1). WMR’s results were consistent with IVW (*P* = .9658; OR = 1.01; 95% CI, 0.59–1.71) (Table 1). In addition, there was no pleiotropy for these selected instrumental variables according to Egger’s intercept. We also performed the MR analysis between BMI and NAFLD from histology. The 688 SNPs associated with BMI were used as the instrumental variables. According to the IVW analysis (*P* = .0907; OR = 1.03; 95% CI, 0.99–1.07), WMR analysis (*P* = .462; OR = 1.04; 95% CI, 0.94–1.15), and MR Egger’s intercept, there was no association between BMI and NAFLD (Table 2).

**Table 1:** Results of Mendelian randomization study with NAFLD (EMERGE network) as outcome.

	N (SNP)	IVW		MWR		MR Egger	
		Odds ratio (95% CI)	*P* value	Odds ratio (95% CI)	*P* value	Odds ratio (95% CI)	*P* value
Obesity indexes							
BMI	690	0.94 (0.68, 1.31)	.7356	1.01 (0.59, 1.71)	.9658	0.65 (0.31, 1.37)	.2606
WHR	364	0.82 (0.53, 1.28)	.3868	0.94 (0.49, 1.80)	.8605	0.95 (0.31, 2.88)	.9317
Diabetes indexes							
FG	29	1.13 (0.52, 2.45)	.7561	1.29 (0.39, 4.26)	.6754	1.2 (0.23, 6.42)	.8167
FI	10	0.42 (0.01, 12.06)	.6121	0.37 (0.01, 9.77)	.5541	0.01 (0.00, 5.64)	.2760
HbA1c	40	0.44 (0.20, 0.97)	.0437	0.33 (0.06, 1.66)	.1787	0.39 (0.05, 2.97)	.3655
Inflammation index							
CRP	36	1.19 (0.94, 1.50)	.1325	1.21 (0.92, 1.57)	.1691	1.90 (0.94, 3.81)	.0729

The *P* value of the intercept term in all results obtained by Egger’s method was greater than .05, i.e., not significant.

BMI = body mass index, CRP = C-reactive protein, FG = fasting blood glucose, FI = fasting insulin, HbA1c = glycosylated hemoglobin, IVW = inverse variance weighted method, MWR = weighted median method, NAFLD = non-alcohol fatty liver disease, SNP = single nucleotide polymorphism, WHR = waist-to-hip ratio.

**Table 2:** Results of Mendelian randomization study with NAFLD (histologic) as outcome.

	N(SNP)	IVW		MWR		MR Egger	
		Odds ratio (95% CI)	*P* value	Odds ratio (95% CI)	*P* value	Odds ratio (95% CI)	*P* value
Obesity indexes							
BMI	688	1.03 (0.99, 1.07)	.0907	1.04 (0.94, 1.15)	.4616	0.99 (0.73, 1.36)	.9984
WHR	370	1.008 (1.002, 1.013)	.0078	1.01 (0.97, 1.05)	.6555	1.02 (0.84, 1.23)	.8230
Diabetes indexes							
FG	28	1.20 (0.64, 2.29)	.5624	1.47 (0.59, 3.67)	.4051	1.03 (0.29, 3.63)	.9532
FI	9	0.19 (0.03, 1.51)	.1170	0.64 (0.10, 4.06)	.4552	0.01 (0.00, 5.58)	.2139
HbA1c	42	0.34 (0.09, 1.27)	.5721	1.19 (0.47, 3.06)	.7044	0.34 (0.09, 1.27)	.1088
Inflammation index							
CRP	34	0.99 (0.95, 1.04)	.7685	0.97 (0.90, 1.05)	.5125	0.95 (0.75, 1.19)	.6765

The *P* value of the intercept term in all results obtained by Egger’s method was greater than .05, i.e., not significant.

BMI = body mass index, CRP = C-reactive protein, FG = fasting blood glucose, FI = fasting insulin, HbA1c = glycosylated hemoglobin, IVW = inverse variance weighted method, MWR = weighted median method, NAFLD = non-alcohol fatty liver disease, SNP = single nucleotide polymorphism, WHR = waist-to-hip ratio.

For WHR, we used 364 genetic variants to perform the MR study between WHR and NAFLD from eMERGE network. The results for the IVW method (*P* = .3868; OR = 0.82; 95% CI, 0.53–1.28) and WMR method (*P* = .8605; OR = 0.94; 95% CI, 0.49–1.80) were nonsignificant (Table 1). The intercept in MR Egger indicated that there was no potential pleiotropy of our selected SNPs. We also performed the MR study between WHR and NAFLD from histology. 370 SNPs were selected as the instrumental variables for this MR analysis, the result for IVW method (*P* = .0078*; OR = 1.008; 95% CI, 1.002–1.013) and the results of MR Egger showed that there was no pleiotropy in instrumental variables, which indicated that there was a potential causal association between WHR and NAFLD (Table 2).

### 3.2. Mendelian randomization analyses between blood glucose related indicators and nonalcoholic fatty liver disease

We used 29 genetic variants as instrumental variables in MR analysis between FG and NAFLD from eMREGE network. The results for the IVW and WMR method were *P* = .7561; OR = 1.13; 95% CI, 0.52–2.45 and *P* = .6754; OR = 1.29; 95% CI, 0.39–4.26, respectively (Table 1). In addition, Egger’s intercept showed that there was no potential pleiotropy. The second NAFLD datasets were used as outcome to perform the MR analysis, the results for IVW method (*P* = .5624; OR = 1.20; 95% CI, 0.64–2.29) and MWR method (*P* = .4051; OR = 1.47; 95% CI, 0.59–3.67) were also insignificant. The Egger’s result showed that there was no pleiotropy in instrumental variables (Table 2). From above, there was no causal association between FG and NAFLD.

For FI, we used 10 SNPs in MR analysis between FI and the first NAFLD dataset. The IVW method (*P* = .6121; OR = 0.42; 95% CI, 0.01–12.06) showed no causal association between FI and NAFLD (Table 1). The MWR results (*P* = .5541; OR = 0.37; 95% CI, 0.01–9.77) were consistent with IVW method. And MR Egger results showed no pleiotropy in this analysis (Table 1). Then, 9 SNPs were used as instrumental variables with which to perform the MR analysis between FI and the second NAFLD dataset. The results for IVW method (*P* = .1170; OR = 0.19; 95% CI, 0.03–1.51) and MWR method (*P* = .4552; OR = 0.64; 95% CI, 0.10–4.06) showed that FI has no causal effect on NAFLD (Table 2). There was no pleiotropy SNP from the results for MR Egger, there was no causal relationship between FI and NAFLD.

For HbA1c, we used 40 SNPs associated with HbA1c as instrumental variables in MR study between HbA1c and NAFLD from eMERGE network. The result for IVW method showed that HbA1c had a causal effect on NAFLD (*P* = .0437*; OR = 0.44; 95% CI, 0.20–0.97), and Egger’s intercept showed there was no pleiotropy in these SNPs (Table 1).

### 3.3. Mendelian randomization analyses between C-reactive protein and nonalcoholic fatty liver disease

We used 36 genetic variants in MR analysis between CRP and NNAFLD. According to the results for IVW (*P* = .1325; OR = 1.19; 95% CI, 0.94–1.50) and MWR (*P* = .1691; OR = 1.21; 95% CI, 0.92–1.57) method from the analysis on the first NAFLD datasets showed that CRP had not causal effect on NAFLD (Table 1). From analysis on the second NAFLD datasets, the results for IVW (*P* = .7685; OR = 0.99; 95% CI, 0.95–1.04) and MWR (*P* = .5125; OR = 0.97; 95% CI, 0.90–1.05) method were consistent with the first one (Table 2). They both showed there was no pleiotropy SNPs, indicating that CRP had not causal effect on NAFLD.

## 4. Discussion

This is the first large-scale study to simultaneously delineate the causal relationships between several clinical indicators (including BMI, WHR, FG, FI, HbA1c, and CRP) and NAFLD using MR analysis. Overall, we found that the genetically driven WHR and HbA1c were causal factors for NAFLD, while BMI, FG, FI, and CRP were not causal factors for NAFLD. Our finding hence revealed new insights into how nature and nurture underpin diseases and NAFLD, providing positive effect on the causes and prevention of these diseases.

Our study interested an exposure obesity which was a prevalent issue in the world, aiming to reveal the relationship between obesity and NAFLD. Obesity was highly associated with NAFLD in observational study.^[41]^ For example, a recent research in a Taiwanese population showed that both male and female participants with high BMI and WHR were significantly associated with NAFLD.^[14]^ Genetically WHR was proved to be a causal risk factor for NAFLD, while genetically BMI was proved to be not a causal risk factor for NAFLD in our study, which might attribute to the differences in study population ethnicity and sample size of the study population. In addition, there may be a reverse causal relationship between BMI and NAFLD, i.e., genetically NAFLD may induces increasing in BMI. For example, the most prominent genetic risk factor for NAFLD is the SNP that causes the I148M substitution (rs738409) in the patatin-like phospholipase domain-containing protein 3 (PNPLA3), whose function in inducing NAFLD was independent to obesity.^[42]^ Furthermore, the presence of hepatic steatosis in NAFLD patients could contribute to the metabolic dysfunction of liver cell, which might induce the accumulation intrahepatic triglyceride and exacerbate obesity.^[43,44]^ Actually, HbA1C was proved to be a more reliable biomarker in predicting the occurrence and severity of NAFLD compared with BMI, and waist circumference, which was consistent with our study.^[45]^

Xie et al showed that NAFLD was closely related with the HbA1c level, and the HbA1c level was positively associated to the development of NAFLD in non-diabetic American population.^[46]^ In addition, the increasing level of HbA1c was positively correlated to the severity of liver steatosis in subjects with prediabetes, indicating that necessity of routinely monitoring the HbA1c level in patients with prediabetes. While the Jee’s study demonstrated the increased level of HbA1c independently elevated the risk of incident NAFLD in population with prediabetes and normal glucose tolerance, not in the patients with diabetes.^[47]^ The increased level of HbA1c could stimulate the receptor for advanced glycation end products, promote hypoxia and suppress NO release, resulting in the progression of NAFLD,^[48]^ which might explain the underlying mechanism for the risk factor of HbA1c in NAFLD. FG and FI were considered to be one of the prognostic indicators of NAFLD in the clinical practice.^[49]^

Although FG and FI were represented as associated factors of NAFLD in previous studies, no causal relationship was observed between FG, FI and NAFLD in our study. Another MR analysis pointed that NAFLD could be significantly elevated by the genetically obesity and diabetes, but their causal relationships in FG, FI and NAFLD were not robust because of the inconsistent results of sensitivity analysis.^[50]^ In our study, we used the largest and latest GWAS summary data, and our SNP screening process is more rigorous by adding the F statistics criterion. In our analysis, genetically WHR and HbA1c were associated with NAFLD.

Taking the results that the HbA1c level and WHR are correlated with the risk of NAFLD, it is necessary to carry out efficient measures to adjust these 2 factors to avoid the occurrence and development of NAFLD. It was reported that educational intervention on lifestyle could effectively help the NAFLD patients control their WHR, which was an effective and cost-effective strategy for the treatment of NAFLD.^[51]^ In addition, the dietary advice (including intermittent calorie restriction and low-carb high-fat diet) were proved to be useful in the control of the BMI and WHR for patients with NAFLD, bringing benefit to the NAFLD patients.^[52]^ Based on a randomized controlled trial, NAFLD patients who had low-carbohydrate, high-fat diet demonstrated a decrease of 6.1 mmol/mol HbA1c (95% CI, 30–9.2 mmol/mol) than patients had high-carbohydrate, low-fat diet, and this diet could reduce hepatic steatosis in NAFLD patients.^[53]^ Therefore, specifying a personalized and reasonable dietary plan is crucial for the treatment of NFALD patients.

This study has several strengths. First, to the best of our knowledge, it is the first time to use MR analysis to evaluate the causal relationship between the 6 clinical indicators (including BMI, WHR, FG, FI, HbA1c, and CRP) and NAFLD. Compared with clinical observational studies, the MR analysis has the advantage of circumventing the impact of potential reverse causation and confounding factors. Second, summary data were obtained from the latest and largest available GWAS meta-analysis, and thus the result of our analyses was not susceptible to bias caused by data selection. Finally, the MR-PRESSO and Q statistics were performed to reduce potential pleiotropy and heterogeneity, which further validated the reliability and stability of this MR analysis.

Nonetheless, there were also certain limitations in this study. Due to the limited data access and insufficient clinical characteristics of participants, summary data was not analyzed on the basis of population stratification. In addition, our study demonstrated the potential causality between WHR and HbA1c with NAFLD. However, further extensive researches are needed to confirm this definite causality and validate the result of MR analysis.

## 5. Conclusion

To sum up, our study suggested that WHR and HbA1c might be potential causal factors for NAFLD based on MR analysis, indicating that WHR and HbA1c could be potential indicators in the clinical treatment of patients with NAFLD.

## Author contributions

**Data curation:** Xing Wang, Lichao Cheng, Chao Geng.

**Formal analysis:** Lichao Cheng, Chao Geng.

**Investigation:** Chao Geng.

**Methodology:** Xing Wang, Jian Li, Chao Geng.

**Project administration:** Jing Gao, Jian Li.

**Resources:** Xing Wang, Jing Gao, Jian Li.

**Software:** Jing Gao, Jian Li, Chao Geng.

**Supervision:** Dianpeng Zhao.

**Validation:** Dianpeng Zhao, Chao Geng.

**Visualization:** Dianpeng Zhao, Lichao Cheng, Chao Geng.

**Writing – original draft:** Chao Geng.

**Writing – review & editing:** Dianpeng Zhao.
